# Food Avoidance beyond the Gluten-Free Diet and the Association with Quality of Life and Eating Attitudes and Behaviors in Adults with Celiac Disease

**DOI:** 10.3390/nu16193411

**Published:** 2024-10-08

**Authors:** Anne R. Lee, Patricia Zybert, Zhijun Chen, Jessica Lebovits, Randi L. Wolf, Benjamin Lebwohl, Peter H. R. Green

**Affiliations:** 1 Celiac Disease Center at Columbia University, New York, NY 10032, USA; jl4877@cumc.columbia.edu (J.L.); bl114@cumc.columbia.edu (B.L.); pg11@cumc.columbia.edu (P.H.R.G.); 2 Program in Nutrition, Department of Health Studies & Applied Educational Psychology, Teachers College, Columbia University, New York, NY 10027, USA; paz4@tc.columbia.edu (P.Z.); zc2530@tc.columbia.edu (Z.C.); wolf@tc.columbia.edu (R.L.W.)

**Keywords:** celiac disease, food avoidance, eating patterns, gluten-free diet

## Abstract

Background: The only treatment for Celiac Disease (CeD), which affects about 1% of the population, is a gluten-free diet (GFD). Studies have indicated an association between the GFD, a diminished quality of life (QOL), and maladaptive eating patterns. This study aims to explore food avoidance behaviors in adults with CeD. Methods: This cross-sectional study assessed 50 adults with biopsy-confirmed CeD who completed validated surveys evaluating demographics, psychological factors, QOL, eating pathology, and food avoidance. Results: Overall CDQOL scores were good (mean: 62.7 out of 100). However, 58.0% of the participants self-elected to avoid one or more additional foods without diagnosed allergies or intolerances. Those avoiding one or more other foods had lower QOL scores (57.4 (23.2) vs. 70.2 (15.9)) compared to those only avoiding gluten (*p* = 0.034). The mean depression score (CESD) for the group avoiding foods beyond gluten was in the depressive range, unlike those avoiding only gluten (16.0 (4.9) vs. 13.6 (4.0), *p* = 0.078), with 77% of those avoiding more than gluten scoring above the CESD cut-off point of 15, indicating clinical depression. Conclusions: Over half of participants (58%) reported avoiding additional foods beyond the GFD, a behavior associated with decreased QOL and increased depression.

## 1. Introduction

### Background

Celiac Disease (CeD) is a genetically mediated autoimmune response triggered by dietary gluten and causing damage to the mucosal lining of the small intestine as well as systemic symptoms [1]. CeD affects multiple systems in the body and can manifest with a variety of symptoms and health problems [1,2]. Exposure to gluten in an individual with CeD causes malabsorption of nutrients in the small intestine, which can lead to vitamin and mineral deficiencies and, in turn, to other chronic conditions such as bone loss, neuropathy, metabolic conditions, infertility, and neuropsychiatric symptoms [3,4,5]. The prevalence of CeD is about 1% of the United States population [2]. This has increased up to 5-fold in the United States since 1950 and rates continue to rise due to both increased awareness and improved testing [6,7].

Currently, the only treatment for CeD is lifelong adherence to the gluten-free diet (GFD) [6,8]. Once a strict GFD is initiated, the immune response abates and the intestines heal, resulting in clinical, serologic, and histologic improvement [9,10,11]. However, maintaining a strict GFD requires daily diligence. Shah et al. discussed that the patient-reported treatment burden of CeD was rated second only to that of end-stage renal disease [12].

Quality of life (QOL), which is the overall sense of wellbeing [13,14] of an individual with CeD, typically improves post-diagnosis and with the initiation of the GFD [13,14]. After the initial improvement, the social domain appears to decline due to the restrictive GFD [13]. The chronic nature of the disease, as well as the persistent vigilance needed to maintain a GFD, has been reported as a major factor in the diminished QOL scores in this population [13,14,15,16,17]. Lee et al. [13] reported a significant negative impact on the social domain of QOL associated with the GFD, specifically dining out, social events, and travel in adults. In the study by Cranney et al. [14], individuals with CeD dramatically changed their social behaviors after starting the GFD. Ninety-four percent brought their own food when traveling or dining out, eighty-one percent no longer dined out, and thirty-eight percent avoided travel. Participants in a qualitative study [18] reported having concerns over food safety, feeling isolated, and always thinking about their food.

Eating disorders (EDs) are often associated with anxiety and alterations in mood, as well as physical complications, such as gastrointestinal (GI) symptoms, hormonal disturbances, and even heart disease [19]. Most ED patients report fullness, abdominal distention, and GI discomfort [19]. Recent studies have investigated associations between CeD and ED [20,21,22]. One study [21] found that women with CeD were 46% more likely to develop anorexia nervosa than their population-based sex- and age-matched controls. A previous anorexia nervosa diagnosis was associated with CeD, suggesting a bi-directional relationship between CeD and ED [23]. Satherley hypothesized that some individuals have a great deal of distress in response to the weight gain often associated with CeD diagnosis and implementation of the GFD, which may result in disordered eating behaviors such as purposeful gluten ingestion or restrictive or bulimic eating behaviors [24].

In addition to the GFD, individuals with CeD may restrict their intake of a variety of foods prior to diagnosis in an attempt to identify triggers for their symptoms. For example, the prevalence of lactose intolerance is thought to be 10% in individuals with CeD and increases to 50% in those with malabsorption [25]. As some digestive enzymes, such as lactase, reside at the top of the intestinal villi, avoiding dairy products may decrease symptoms in individuals with CeD when damage is present. Avoiding fiber-rich foods such as oats or foods high in fermentable carbohydrates may generally decrease symptoms such as bloating, as many of these foods may induce GI symptoms in patients. However, for the majority of individuals with CeD, the initiation of a GFD is associated with resolution of their symptoms. Counseling with a CeD specialist dietitian will provide a medically appropriate individualized approach to dietary management.

The potential for ED development is present in CeD, as well as other GI disorders, due to subtle but pervasive maladaptive eating behaviors associated with restrictive diet therapies [26]. These behaviors include fasting, excessive physical activity, hypervigilance, and preoccupation with meals and food preparation. These, in turn, may disrupt daily life, manifesting as avoidance of social events, dining out, and travel, and may even promote social isolation [27]. Improvement or even cessation of symptoms on the GFD due to hypervigilant adherence may be linked to increased anxiety associated with chance exposure, food preparation, ingredients, and food labeling and may be precursors to maladaptive eating behaviors and possibly ED [18,23,25,27,28,29]. Satherley and colleagues [30] noted that strict GFD adherence, increased stress, anxiety, and an unhealthy relationship with food were predictors of maladaptive eating behaviors. In a study of adolescents with CeD, being overweight, older, and female was associated with disordered eating behaviors [30]. It is imperative to be aware of the impact of the GFD and disease in individuals with CeD to prevent the escalation of maladaptive eating behaviors into clinical ED. The aim of this study is to investigate eating patterns in adults with CeD that may suggest maladaptive eating behaviors, specifically focusing on food avoidance beyond the GFD and its relationship with quality of life (QOL), depression, anxiety, eating pathology, food attitudes, and preoccupation with food. We seek to understand how these food avoidance behaviors are linked to psychological wellbeing and the development of maladaptive eating behaviors in this population.

## 2. Materials and Methods

The current investigation was a secondary investigation in a cross-sectional study of 50 adults with CeD conducted in 2021. The aim of the original study [31] was to investigate the prevalence of EDs and disordered food attitudes and beliefs in a sample of adults with CeD. Participants were asked to complete a series of self-report surveys. In this investigation, we focused on food avoidance.

### 2.1. Recruitment

Consecutive patients with who visited the Celiac Disease Center of Columbia University for a regularly scheduled clinic appointment were invited to participate. Inclusion criteria included ≥ 18 years of age, biopsy-proven CeD, on the GFD for at least one year, and no prior or current diagnosis of an ED. Details of the study protocol and sample have been reported elsewhere [31].

### 2.2. Study Measures

The following demographic data were collected: gender, ethnicity, race, marital status, level of education, household income, age at enrollment, years since CeD diagnosis, height, and weight. Validated surveys measured CeD-related GI symptoms (Celiac Disease Symptom Diary [CDSD]) [32], adherence to the GFD (Celiac Disease Adherence Test [CDAT]) [32], CeD-specific QOL (CD-QOL) [33], depression (Center for Epidemiologic Studies Depressive Scale [CESD]) [34], anxiety (State and Trait Anxiety Inventory [STAI]) [35], Eating Pathology Symptoms Index (EPSI) [36], and food attitudes and behaviors (Celiac Disease Food Attitudes and Behaviors [CDFAB]) [37].

Additionally, we collected data on food avoidance beyond the GFD. A series of questions designed specifically for this study queried whether participants avoided fifteen foods (dairy/casein/milk protein, eggs, fish, lactose, fructose, oats, potatoes, peanuts, quinoa, sesame, shellfish, soy sugar, tree nuts, all grains), who advised them to avoid the food (e.g., physician, dietitian, functional medicine practitioner, self), and whether they have a formal diagnosis of an allergy to that food. The questionnaire also asked about specific dietary patterns (kosher, Low Fermentable Oligosaccharides, Disaccharides, Monosaccharides and Polyols (FODMAP), paleo, vegan, vegetarian, pescatarian, keto, other), other specific foods they chose to avoid, and why. Finally, using a six-point Likert scale from never to always, we queried how often participants thought about food and/or eating and how often they thought about their weight.

### 2.3. Statistical Analysis

Using the specific protocol for each instrument, the total and sub-scores for each survey tool were calculated. The sample demographics and health characteristics were analyzed using descriptive statistics with mean (SD) presented for continuous variables and N (%) for categorical variables. For additional analysis, participants were grouped by the presence/absence of additional foods avoided beyond gluten. Groups were compared using independent sample t-tests and correlations using chi square. Statistical analysis was performed using SPSS software version is 29, with *p* < 0.05 2-sided considered statistically significant. 

### 2.4. Ethical Approval

The Institutional Review Boards at Columbia University Medical Center (AAAS5501) and Teachers College (19-479) approved this study. Written consent was obtained at the time of enrollment.

## 3. Results

As we described in previous publication, the study cohort comprised 50 adult participants, whose demographics are described in Table 1. The sample was predominately female (70%), white (94%), and with college or higher education (84%). The mean age of the sample was 29.6 (SD = 7.4) years. The mean body mass index (BMI) was 23.2 kg/m^2^ (4.0), which is within the normal range, with the participants categorized as follows: 2 (4%) were obese, 11 (22%) were overweight, 32 (64%) had normal weight, and 5 (10%) were considered underweight.

### 3.1. Overall Survey Results

The mean CDAT adherence score for the total sample was 11.9 (SD = 3.3), with scores greater than 13 suggesting inadequate adherence to the GFD. Twenty-eight percent of participants would be classified as nonadherent with a score over 13. The mean CD-QOL score for the total sample was 62.7 (SD = 21.2) out of 100, with higher scores associated with better QOL. The mean CESD depression scale was 15.0 (SD = 4.6). The clinical cut point for diagnosis of depression is >15, and thirteen (26%) of the participants scored over 15 on the CESD scale. The mean state anxiety score was 37.4 (SDS = 13.1) and the mean trait anxiety score was 39.8 (SD = 10.7). A STAI score above 39 meets the cut point for clinical anxiety. Of the participants, 40% would be considered in an anxious state and 44% would be considered to have anxious traits.

### 3.2. Avoiding Food

Table 2 presents result of the comparisons of those who reported avoiding 0 versus 1+ other foods. There was no difference in age at enrollment between the groups. However, there were differences based on length of time since diagnosis. Those avoiding more than gluten tended to have been diagnosed with CeD more recently (6.0 (4.5) vs. 8.9 (6.0) years, *p* = 0.060). There were no differences between the groups in terms of BMI, number of GI symptoms (i.e., CDSD), or adherence to the GFD (i.e., CDAT). Those individuals avoiding no other foods had a lower mean number of other dietary patterns followed (0.1 (0.3) vs. 0.9 (1.1), *p* = 0.002).

### 3.3. Avoiding Food and Specific Variables

Twenty-nine of the fifty (58.0%) participants avoided one or more of the 15 foods listed. Oats and dairy/casein/milk protein were the most frequently avoided, each being avoided by 11 (22.0%) participants. Other specific foods avoided included lactose (n = 10, 20%), sugar (n = 8, 16%), soy (n = 6, 12%), eggs (n = 5, 10%), and fructose (n = 3, 6%). In all, there were 71 instances of foods avoided. Only about a third (36.6%) of the foods avoided were avoided on the advice of a physician and/or dietitian. Almost half of the foods (49.3%) were avoided by self-evaluation and/or online information (not dietitian nor physician). Only a fifth (21.1%) of avoidances were accompanied by a formal diagnosis of allergy.

In addition to avoiding specific foods, we found a third of the subjects (32.0%) reported following one or more other dietary patterns. The most reported patterns were kosher (n = 7, 14.0%) and vegetarian (n = 5, 10.0%). Other dietary patterns included anti-inflammatory (n = 4, 8%), vegan (n = 3, 6%), low-FODMAP (n = 2, 4%), and paleo (n = 2, 4%). In addition to following a GFD, eight subjects (32.0%) adhered to at least one additional dietary pattern.

**Figure 1:**
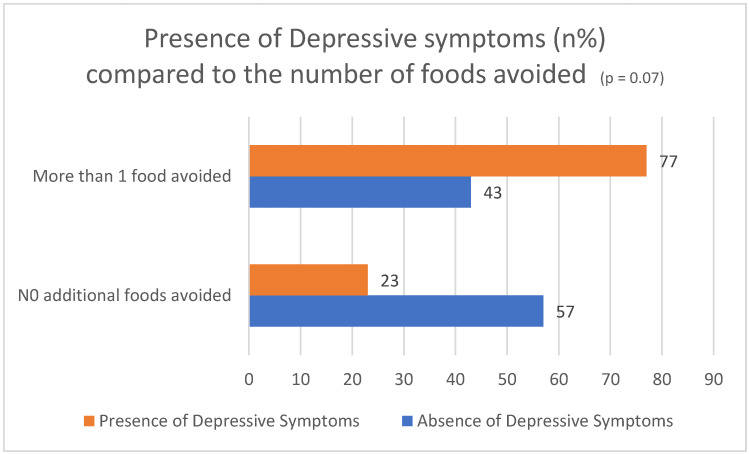
Percentage of participants meeting the cut point for depressive symptoms, comparing those avoiding more than gluten with those just avoiding gluten.

Interestingly, those who did not avoid other foods apart from gluten had higher mean overall QOL scores, as well as higher scores in some QOL subscale scores (overall CD-QOL 70.2 (15.9) vs. 57.4 (23.2), *p* = 0.034). The overall depression scores (CESD) were not statistically significantly different between those who avoided no other foods and those avoiding more than 1+. However, the mean for the group avoiding foods in addition to gluten was in the range suggesting depression, a score > 15, while that for the group avoiding just gluten was not (13.6 (4.0) vs. 16.0 (4.9), *p* = 0.078 (Figure 1)).

Both state and trait anxiety scores were lower, indicating a less anxious state and fewer anxious traits for those avoiding no other foods (32.0 (11.6) vs. 41.2 (12.9), *p* = 0.012 and 36.2 (9.2) vs. 42.3 (11.1), *p* = 0.043, respectively).

Two subscales of the EPSI, body dissatisfaction (7.3 (7.1) vs. 11.7 (7.9), *p* = 0.052) and restricting (3.1 (4.7) vs. 6.1 (5.5), *p* = 0.053), had nearly significant differences between those avoiding no additional foods vs. those avoiding more than just gluten. There was a significant difference in the EPSI body dissatisfaction scores between those who avoided more foods and those who did not, with the higher body dissatisfaction scores associated with those who avoided more foods.

### 3.4. Thinking about Weight

The CDFAB is an 11-item CeD-specific tool used to assess food attitudes and behaviors using a 5-point Likert scale ranging from strongly disagree to strongly agree. A total higher score suggests more maladaptive eating patterns. The total score encompasses subscales of fear response, adaptive response, and food attitudes, querying beliefs on food contamination, food safety, and risk taking. When we queried how often participants thought about their weight, we found differences in food attitudes on the adaptive response subscales of the CDFAB. Roughly a third (32%) reported thinking about their weight “often”, “almost always”, or “always”. In the CD-FAB adaptive response subscale score, only the group that thought about their weight “sometimes” and “often” vs. “almost never” and “sometimes” was associated with higher scores indicating more maladaptive eating behaviors (12.4 (5.5) vs. 15.7 (6.0), *p* = 0.055). Those in the group avoiding 1+ other foods reported thinking about their weight more often than those avoiding no other foods (means (SD) on a 6-point scale from 0 to 5; 2.6 (1.1) vs. 1.8 (1.1), *p* = 0.013. Higher (worse) CESD depression scores were also associated with thinking about weight more often (r = 0.288, *p* = 0.043). The decreased dietary adherence, decreased QOL, increased anxiety, and depression scores may impact intake, eating patterns, and avoidance of foods.

### 3.5. Thinking about Food

A majority of participants (64%) reported thinking about eating or food “often”, “almost always”, or “always”. Increased frequency of thinking about food and/or eating was significantly associated with several of the outcome variables measured, including higher (worse) CDAT scores and QOL (r = −0.595, *p* < 0.001), and the dysphoria QOL subscale (r = −0.388, *p* = 0.005), limitations QOL subscale (r = −0.638, *p* < 0.001), health concerns QOL subscale (r = −0.531, *p* < 0.001), and inadequate treatment QOL subscale (r = −0.202, *p* = 0.159) were negatively impacted. Thinking about food and/or eating more often was associated with higher (worse) STAI anxiety state (r = 0.380, *p* = 0.007) and STAI anxiety trait (r = 0.409, *p* = 0.003) scores. Thinking about food and/or eating was associated with higher (worse) CESD depression scores (r = 0.327, *p* = 0.021).

## 4. Discussions

More than half of the participants reported avoiding one or more foods beyond the GFD. This additional food avoidance was associated with decreased QOL and increased anxiety and depression. Avoidance was not associated with better GFD adherence as measured by the CDAT. Nearly two-thirds of participants reported thinking about eating and/or food “often”, “almost always”, or “always”. Thinking more often about food and/or eating was associated with several negative measures, including decreased QOL and dietary adherence and increased anxiety and increased depression. Those individuals that avoided more than just gluten also thought about their weight more often than those just avoiding gluten. This may further exacerbate restrictive or maladaptive food behaviors. In the study by Latzer et al. [38], increased disordered eating behaviors (DEBs) were significantly associated with those participants who were overweight, older age, and female. In fact, 17% of the female participants in the study exhibited disordered eating behaviors (DEBs) [38]. In this study, we also found increased rates of depression and anxiety associated with increased dietary restrictions or avoiding additional foods to gluten. We also found a third of the subjects were following one or more dietary patterns in addition to their prescribed GFD. It would be important to investigate the impact of these dietary patterns on QOL, ideally identifying whether the dietary pattern framework enhanced adherence and diminished anxiety or increased anxiety and diminished overall QOL.

Perseveration of food may also highlight a dissonance between perception of and actual GFD adherence. While attentiveness to food is a necessity for those with CeD, there may be a point where attentiveness becomes counterproductive. Thinking about food and/or eating more often did not increase GFD adherence but did increase anxiety measures.

An earlier study by Lee and colleagues [13,39] found that social situations negatively impacted GFD adherence. Both males and females reported a high rate of dietary compliance initially (98% each for males and females). When probed further, a surprising number admitted to dietary indiscretion and intentional gluten ingestion when dining out. In contrast to the study from Lebovits et al. [40], females reported a slightly higher degree of intentional noncompliance than males (88% vs. 81% at social activities, 88% vs. 82% in restaurants, and 67% vs. 58% with friends). Almost three-quarters of participants (73%) reported the main reason for their dietary indiscretion was the restrictive nature of the GFD [40]. Sixty-nine percent reported being uncomfortable with explaining their dietary needs in a social situation [40]. These findings indicate the high degree of discomfort associated with maintaining GFD in a social setting, which raises the question of how this increased negative attention, social anxiety, and discomfort may impact one’s eating pattern potentially limiting, avoiding, or further restricting intake beyond just gluten.

Anxiety and depression levels in our study population were high, with total group means equaling or closely approaching clinical cut-off points. Means for those avoiding more than just gluten exceeded those clinical cut points. A study by Joelson and colleagues found that the correlation between symptoms and adherence varied according to whether depression was also present [41]. In the study, 46% of the participants felt depressed due to the burden of their disease [41]. The question is this: is the increased burden of GFD adherence at the root of anxiety and depression in a proportion of individuals, or do depression and anxiety promote more restrictive behavior and perseveration around food? These findings indicate the high degree of discomfort associated with maintaining a GFD in a social setting, which raises the question of how this increased negative attention, social anxiety, and discomfort may impact one’s eating pattern potentially limiting, avoiding, or further restricting intake beyond just gluten. The intersection of depression with avoidance of foods beyond the GFD and the preoccupation with eating and/or food warrants further investigation.

Recent studies have associated diminished QOL with maladaptive approaches in management of the GFD. The study by Cadenhead et al. [17] describes eating patterns in adults with CeD that suggest maladaptive eating behaviors. In the Cadenhead study [17], which assessed approaches to GFD management based on four dimensions—flexibility (vs. rigidity), trust (vs. avoidance), confidence (vs. controlling behavior) and awareness (vs. preoccupation with)—approximately half of the study sample (53.3%) were classified as having more maladaptive approaches to maintaining a GFD compared to the other participants. Maladaptive approaches were associated with decreased QOL but not with increased GFD adherence. The authors found that extremely vigilant adults had lower overall QOL scores than less vigilant adults (64.2 (16.0) vs. 77.2 (12.2)). Lee et al. [39], using the eating behavior survey CDFAB, found that participants aged 23–35 with higher (worse) CDFAB scores had the highest social anxiety scores (SAQ).

We do not know why an individual patient may choose to avoid more foods than gluten. However, participants in this study who avoided foods beyond the GFD tended to have been diagnosed more recently than those who were diagnosed longer. For those individuals (36.6%) avoiding foods beyond gluten with the recommendation by either a dietitian or physician, it would be important to determine whether these additional restrictions are long term vs. temporary as the mucosa heals. For individuals who need to avoid multiple foods (e.g., gluten, dairy, etc.), it is essential that these restrictions are managed and counseled in a healthy way, rather than in a maladaptive or hypervigilant manner, to maximize quality of life (QOL). It could be speculated that with ongoing dietary counseling, individuals with CeD may be less inclined to self-limit their food choices beyond the already-restrictive GFD. In addition, further testing may confirm the need for additional food class restrictions, such as a need to restrict lactose or fructose for specific carbohydrate intolerances. Counseling with a dietitian specializing in CeD may also serve to decrease anxiety around the GFD and food availability. Further longitudinal studies are needed to follow patients ensuring the re-introduction of foods initially limited for symptom reduction and the incorporation of a healthy varied intake within the GFD.

### 4.1. Limitations

The small sample size precluded comparisons by reasons for avoiding foods (e.g., advised by physician and/or dietitian vs. self-referral only; presence or absence of formal allergy diagnosis). In addition, the study population was relatively homogenous and atypically affluent and highly educated. Our study population of individuals with CeD, in which symptoms such as bloating and weight gain post-diagnosis due to increased absorption are common, may have limited associations of the Eating Pathology Symptoms Index (EPSI) subscales, which measure body dissatisfaction and concerns with weight. Another limitation is that recruitment was interrupted for several months due to the COVID-19 pandemic. The food avoidance questionnaire was developed for this study and has not been validated elsewhere.

### 4.2. Strengths

The strengths of this study include its prospective design, confirmation of CeD by medical record review, and the use of multiple validated questionnaires. The study population is small and as such is reflective of the center’s patient population. This study provides a novel look at the QOL of individuals on the GFD through the lens of food avoidance and preoccupation with food and/or eating beyond adherence to the GFD.

## 5. Conclusions

In a group already at increased risk of diminished QOL and increased depression, anxiety, and eating pathology symptoms, food avoidance beyond a GFD was found to be associated with adverse outcomes beyond those experienced by those who did not avoid other foods. Food avoidance beyond the GFD may be another warning sign of maladaptive and deleterious eating behaviors, such as avoidant/restrictive food intake disorder (ARFID) (44), among those with CeD. Clinicians need to be aware of the physical and emotional impact of the diet prescription. Ongoing physical symptoms while adhering to the GFD may propel an individual to restrict their intake beyond gluten without appropriate testing or diagnosis. Adherence to a GFD for an individual with CeD is imperative but must be encouraged with mindfulness of the individual’s emotional state. Concern over comorbidities and healing, as well as continued symptoms, may increase anxiety around dining out, chance gluten exposure, or checking ingredients. Fixation on these concerns can lead to hypervigilance which has been associated with more restrictive behavior, potentially leading to further restriction of intake and food. Ongoing clinical management of the individual with CeD must include conversations with the patient to determine their dietary behaviors. Our research has found a large percentage of patients with CeD restrict foods beyond the GFD which might lead to poor psychological outcome or potentially further restrictive behaviors such as ARFID [42]. Further and longitudinal investigation is needed to understand the reasons behind such behavior and its impact on overall health and eating behaviors.

## Figures and Tables

**Table 1:** Demographic of the study population.

Demographics		N (%)
Gender	Female	35 (70.0)
	Male	15 (30.0)
Race	Asian	2 (4.0)
	Black	1 (2.0)
	White	47 (94.0)
Education	Some college	8 (16.0)
	College graduate	32 (64.0)
	Postgraduate	10 (20.0)
Household income	<USD 50,000/year	4 (8.0)
	USD 50–100,000/year	11 (22.0)
	>USD 100,000/year	31 (62)
	Did not disclose	4 (8.0)
		Mean (SD)
Age at enrollment		29.6 (7.4)
Age at diagnosis		22.8 (9.3)
Years since diagnosis		7.2 (5.3)
BMI (kg/m^2^)		23.2 (4.0)

**Table 2:** Comparison of those avoiding 0 foods additional foods beyond those including gluten and those avoiding 1+ such foods.

	0 Foods Avoided N = 21	1+ Foods Avoided N = 29		
	Mean (SD)	Mean (SD)	*t*-Test	*p*
Age at enrollment (yrs)	30.1 (7.2)	29.2 (7.7)	0.4	0.664
Years since CD diagnosis	8.9 (6.0)	6.0 (4.5)	1.9	0.060
BMI (kg/m^2^)	23.5 (4.0)	22.9 (4.1)	0.6	0.559
# GI symptoms past 24 h (CDSD)	1.7 (1.5)	2.1 (1.5)	−0.8	0.405
Gluten-free diet adherence (CDAT)	11.3 (2.6)	12.3 (3.7)	−1.1	0.263
# other dietary patterns followed	0.1 (0.3)	0.9 (1.1)	−3.2	0.002
OVERALL quality of life (CD-QOL)	70.2 (15.9)	57.4 (23.2)	2.2	0.034
Dysphoria subscale	82.7 (20.4)	68.1 (28.5)	2.0	0.050
Limitations subscale	66.8 (17.1)	54.8 (25.1)	1.9	0.064
Health concerns subscale	63.3 (22.2)	52.5 (27.4)	1.5	0.142
Inadequate treatment subscale	77.4 (31.3)	59.5 (32.5)	2.0	0.057
Depression (CESD)	13.6 (4.0)	16.0 (4.9)	−1.8	0.078
Anxiety state (STAI Adults)	32.0 (11.6)	41.2 (12.9)	−2.6	0.012
Anxiety trait (STAI Adults)	36.2 (9.2)	42.3 (11.1)	−2.1	0.043
Body dissatisfaction (EPSI)	7.3 (7.1)	11.7 (7.9)	−2.0	0.052
Binge eating (EPSI)	6.2 (6.1)	9.5 (6.6)	−1.8	0.083
Cognitive restraint (EPSI)	4.0 (2.5)	5.0 (2.9)	−1.3	0.200
Purging (EPSI)	0.1 (0.3)	0.3 (1.0)	−1.1	0.294
Restricting (EPSI)	3.1 (4.7)	6.1 (5.5)	−2.0	0.053
Excessive exercise (EPSI)	5.3 (5.7)	5.8 (4.4)	−0.3	0.734
Negative attitude toward obesity (EPSI)	3.2 (4.8)	3.9 (5.0)	−0.5	0.636
Muscle building (EPSI)	1.7 (2.6)	2.5 (3.0)	−1.0	0.299
CD-FAB TOTAL	33.0 (13.7)	39.9 (16.0)	−1.6	0.116
Food attitude subscale	13.1 (7.3)	15.4 (7.4)	−1.1	0.271
Fear response subscale	7.4 (3.4)	8.7 (4.5)	−1.1	0.274
Adaptive response subscale	12.4 (5.5)	15.7 (6.0)	−2.0	0.055
Thinking about eating and/or food	2.7 (0.8)	3.2 (1.0)	−1.9	0.068
Thinking about your weight	1.8 (1.1)	2.6 (1.1)	−2.6	0.013

## Data Availability

The original contributions presented in the study are included in the article, further inquiries can be directed to the corresponding author.
